# Supplementary material for: Sparse connectivity for MAP inference in linear models using sister mitral cells
Source: PLoS Comput Biol. 2022 Jan 31;18(1):e1009808. doi: 10.1371/journal.pcbi.1009808 (PMC8830798; doi:10.1371/journal.pcbi.1009808)
Supplement: Attachment — Submitted filename: point-by-point-responses.pdf [file pcbi.1009808.s001.pdf]

Point-by-point reviewer comments and responses

Reviewer #1 (Darío Cuevas Rivera)

General comments
The authors present an extension to existing models for odor encoding/decoding in the brain. It is unclear, however, what the advantages of such a model are and, more importantly, how these advantages can be seen in the simulated results.

I can see two advantages (mentioned by authors) of their model:

(1) Sparsity
Sparsity of connections within the olfactory bulb is mentioned repeatedly and touted as the advantage of the model over previous models. The title itself implies that inference with sparse connections is due to the introduction of sister cells in the model. However, as mentioned multiple times by the authors, the introduction of sister cells does not change the total number of connections. Indeed, given the condition presented in eqn. 14, it is possible to end up with a higher number of connections with sister cells than there was before sister cells, if the blocks are allowed to overlap. In reality, the reduction in the number of connections is achieved by introducing the original mitral cell in the model or, even earlier, by introducing the organization of granule cells into glomeruli.

A natural question is whether the introduction of the mitral cells is necessary at all. Instead, one could simply reduce the connectivity between y and x to form glomeruli. If A_{ij} is a block matrix, the same sparsification is achieved, even in the term A_{ij}A_{ik}x_k. With this, the original equations for the MAP estimate can be used.

The reviewer is correct that it may be possible to find other sparsifying solutions if the matrix A_{ij} has special structure. However, in our work A_{ij} is the affinity matrix, with each element indicating the amount by which a given molecule excites a given glomerulus. We don't have any control over this matrix, therefore we don't assume any special structure, and in this case the implied resulting connectivity is dense. We have updated the text on line 120 to highlight this point.

We should also emphasize that our aim is not to reduce the number of connections *per se*, but to reduce the number of connections that a single neuron in the model has to make. Without special structure in the affinity matrix, and without mitral cells, each granule cell must connect to ~100,000's of others (Fig 1C). Introducing mitral cells reduces the total number of connections, but still requires each mitral cell to connect to ~100,000 granule cells (Fig 1D). Introducing sister cells *and* setting connectivity judiciously reduces the number of granule cells each mitral cell must connect to by a factor of S (Fig 3C, D). It is in this sense that the model sparsifies connectivity, by reducing the number of connections a mitral cell has to make by a factor of S.

Given the lack of experimental support for their model, specifically for the particular usage of the mitral cells suggested by the authors, the best (and perhaps only) way to show that the introduction of the mitral cell is necessary (or at least beneficial) is to show that it enhances inference (accuracy, speed or some other measure) over the simple organization of granule cells into glomeruli. I believe these simulations are necessary for this study if sparsification via mitral cells is to be one of the main selling points of the model.

We fully agree that we need to show that introduction of the sister mitral cells is, at the very least, beneficial. We do that, but for connectivity, not inference: as mentioned in our previous response, sister mitral cells are necessary to reduce connectivity to biologically realistic levels.

(2) Sister cells
The introduction of sister cells allows for a form of noise clean-up, regardless of whether the blocks are overlapping or not. As the authors show, sister cells within a glomerulus could converge to different results, neither of which needs to match the original MAP estimate (i.e. without sister cells), but the average across them does perform MAP (or, equivalently, their introduction of the periglomerular integrator takes care of the same problem). This could be a strong component of the study, but the results seem to suggest that in fact the opposite is true: Figure 10 shows how introducing sister cells in fact detracts from the MAP estimate, increasing RMS.

In our model, without leak, sister cells have no effect on the asymptotic value of MAP inference (although they do affect the transients, as discussed at length in Sec. 2.5). Aside from the likelihood term allowing for the receptor responses to be corrupted by additive noise, we do not have noise in our model and do not add any in our simulations, although that is certainly an important avenue for future research -- it may indeed be the case that sister cells decrease the effect of noise.

Seeing these results, and given my point above about sparsity, what is the advantage of sister cells? Perhaps sister cells do indeed improve inference, and it is the authors' choice of using MAP as a benchmark that creates the results in figure 10, since, as the authors show, MAP itself fails if n > 3 (and perhaps it is not that great even for n =< 3?). Could it be that the non-overlapping sister cells make inference surpass the power of MAP? This could be checked in simulations, by using a different criterion for the success of inference and showing that the model here presented in fact performs better than MAP.

Whether an appropriate usage of sister cells could surpass the power of MAP is a very interesting idea, and it would certainly be an important topic for future work. However, the sole purpose of sister mitral cells in our present work is to reduce the number of connections from mitral cells to granule cells, not the total number of connections, while still performing exact MAP inference. Without sister cells, each mitral cell has to connect to N granule cells to perform MAP. With sister cells, they need only connect to N/S granule cells.

As for the poor performance of MAP for n > 3, this is due simply to the fact that we ran the model using a small number of glomeruli M, typically 50. Increasing M increases the highest value of n that the model can infer (as one would expect from MAP). We have now updated Figure 11 to include results for higher values of M to demonstrate this point.

The above is only a suggestion, but without a result like this, I fail to see how introducing sister cells (over a single mitral cell, as in section 2.1, or over a block connectivity matrix, as I mentioned in (1) above) is beneficial at all.

We apologize for the confusion. We now state clearly at the end of Section 2.2 (lines 221-224) that the advantage of sister cells is to reduce the number of connections from mitral cells to granule cells, by a factor of S.

Major comments

1. (1) Throughout the manuscript, the word “odor” is used to refer to both the total input to the system, and the individual components of the input. The authors should make sure to use different terms for these two concepts and stick to them. For example, they could talk about an odor (total input) and its components, or perhaps an odor and its molecules.

We thank the reviewer for spotting this inconsistency in our usage of the word "odor". In all but one instance (see below) by 'odour' we mean the total input to the system, not the individual components, and we have now updated the text to reflect this, including at the following locations:

- In the first sentence of Section 4.5, “Cortical Readout” (line 826);
- In the caption of Figure 5;
- In the paragraph beginning with "Number of odour components (line 403)";
- Figure 11 caption.

The one exception is in section 4.6, "Application to other models," in which the other model that we refer to does indeed represent the concentration of individual odours, rather than odour components, so we have left that section as is.

2. (2) Where does equation 10a come from? I can only infer, seeing 10b, that \lambda_i = 1 / \sigma ^ 2 (y_i - \sumA_{ij}x_j). However, if this is the case, then the right-hand side of eqn. 10a equals \lambda_i - \lambda_i = 0, which makes no sense. This requires more explanation.

We apologize for the confusion. As we explain starting on line 120, Equation 10a is derived by factoring out the bracketed term in Equation 9 and implementing it with a new variable, lambda_i, whose value at steady state is the bracketed term. This then ensures that the steady state of Equation 10b is the same as Equation 9.

3. In line 151, “It turns out that… doesn’t exactly work”. What does this mean? Did the authors perform the simulations and found the results to be bad? If so, they need to report this. If the authors would prefer not to report this, then the rest of the section (i.e. the introduction of “additional cells and connections”) needs to be justified in detail.

We apologize for the vague phrase "Doesn't exactly work". We meant “Doesn’t yield the correct MAP solution." We have now dropped this, as it mainly added confusion.

4. The section “Cortical readout” should not be part of the Results section. It does not belong to the MAP model (or its extensions), nor do the authors present any results with it. It belongs in the Discussion section.

We thank the reviewer for this point, and we've moved the section "Cortical readout" to the Methods, Section 4.5, and updated the reference in the Discussion accordingly.

5. One of the results is that the MAP estimate reaches equilibrium within a couple hundred of milliseconds, as would be expected from the brain. However, in their simulations, this is determined by the time constants \tau. How were the values for these constants set? Are they biologically plausible? The authors should justify these values.

We thank the reviewer for pointing out this omission. The membrane time constants of the mitral and granule cells were set to be similar to the corresponding charging time constants in (Bhalla and Bower, 1993). For simplicity the time constant of the periglomerular cells was set to be the same as that of the granule cells, and is consistent with e.g. (Tavakoli et al 2018). We have now updated lines 779-782 of 'Section 4.4: Simulations' of the Methods to reflect this.

6. Related to (5), it seems like the leaky integrator converges more quickly than the non-leaky, at least comparing figures 9 and 5, and how oscillations stop earlier on figure 9. If there's a good reason for this, it could be an interesting result on its own.

We thank the reviewer for this observation. The intuitive reason for this is that adding the leak adds a source of dissipation in the system, dampening oscillations. We did not mention it as it's not part of our take-home message, but it may indeed be an interesting question to pursue in future work.

7. In the simulations in section 2.6.2, it is shown that even with a leaky integrator, the error is "low" compared to MAP. However, I see an RMS of a bit under 0.1. Is this actually small? Could the authors provide some reference? How would such RMS impede inference? How does it relate to \sigma?

The reviewer is correct in pointing out the potential ambiguity in terms like "small." However, we believe this term is justified, for two reasons. First, similar to the other figures reporting RMS error results and as indicated by the caption in Figure 5, RMS error results are normalized to their initial, pre-inference values. Hence the reported value of 0.1 is not an absolute term, but means that the leaky inference procedure has still reduced the RMS error by 90%. Secondly, we show in Figure 9 actual inference results for a typical case with RMS of ~0.1. The red triangles in that figure indicate the MAP estimate for the concentrations of components present in the odour (the other concentrations were ~0), and these are all of order 1. The figure shows that the concentrations inferred by the leakly inference procedure as reported in the steady state values of the granule cell activations deviate from the MAP estimate by <0.1. Hence, for these two reasons, we believe we are justified in referring to these errors as "low," or "small."

8. Related to (6), why do the values in figure 10a not match those in 9b? They seem to be an order of magnitude off. If this is a consequence of averaging across many simulations (and even different systems), perhaps error bars would help figure 10a.

We are grateful to the reviewer for spotting this inconsistency. It was due to RMS values in all figures except Figure 10 being normalized to their initial values, as mentioned in the caption for Figure 5D, to allow the reader to determine the magnitude of the reduction in RMS error. In Figure 10 however we were reporting the absolute, un-normalized values for RMS error. We have now updated Figure 10A to use the normalized values, and have updated the caption to emphasize this point. The results in Figures 9b and 10A are now directly comparable, and are similar, as expected by the reviewer.

9. While it is nice to know that the ideas in the paper can be applied to other models, I do not think section 2.7 belongs to the results section, as the authors do not do anything with the resulting equations. This should be in the Discussion section, with the mathematical derivations as supplementary materials. Additionally, the manuscript would benefit from a few other examples for which the ideas of their model could be applied, without necessarily deriving the equations.

We agree with the reviewer and have now moved the section to the Methods, Section 4.6, with a reference in the Discussion.

10. I think the results in the list starting in line 384 is a great inclusion to the manuscript. However, I think the manuscript would read much better if the equations, derivations and figures were sent to the methods and supplementary materials, leaving only the results (the list) and the discussion of the results in the Results section.

Indeed, the 'right' number of equations to include in the Main Text is a difficult question, and we believe, a matter of taste. While the manuscript would be easier to read for some readers if we moved more equations to the Methods, for others it would be harder. Because we are submitting our manuscript to a computational biology journal, we felt that most of our readers would appreciate seeing a little more of the mathematical details in the Main Text.

11. The subsection “Number of odours”, alongside figure 11, does not really explore the features of the model presented in this manuscript. Instead, it focuses on (standard) MAP results. As such, it does not belong in this manuscript. Instead, the interesting question is how the authors’ model deviates from MAP –or even better, from the real cause x*– as n increases.

We thank the reviewer for this point. Before addressing it below, we would like to emphasize that our model does not deviate from MAP inference: in the non-leaky setting, where periglomerular cells are perfect integrators, our model performs MAP inference exactly, as we analytically derive in the text and confirm numerically in e.g. Figures 5 and 6.

The reviewer is correct that panels A and B of Figure 11, which focus on the relation between the number of inferred components and the number of components present in the true odour x*, are standard MAP results. However, the results presented in panel C are specific to our model: in that panel we illustrate the dynamics of inference, and how the model arrives at the MAP solution. As we discuss in the "Linear dynamics" section of Methods, the number of inferred odours, n, influences the dynamics through the Marcenko-Pastur parameter v. That analysis suggested that this effect is limited to the oscillation frequency of the low frequency component of the dynamics ($\xi^{low}_{+/-}$) and leaves the

decay time constant untouched. Panel 11C confirms the latter, by showing that there is little qualitative change in the decay time constant of RMS error towards the MAP solution as the density of the true odour increases. We believe panels A and B in this figure are important because they emphasize the fact that the decay rate is independent of the density of odors even when far more odors are inferred than are present.

12. In the paragraph starting on line 542, the authors make a prediction based on their model. How does the model predict this? In particular, it is the introduction of sister cells in the model that pushes the model away from MAP; how is it that removing this component will move the inference further away from MAP? Additionally, why does the model predict “low amplitude activation of granule cells” when the component is missing?

We are grateful to the reviewer for spotting the lack of justification for our claim about granule cell responses in the absence of periglomerular cells. We would like to point out that as explained above, sister cells do not push the model away from MAP; as derived in the Main Text, the model with periglomerular as perfect integrators implements MAP inference exactly. We have now provided justification for our claim regarding granule cell responses in the Methods Section 4.3, and referred to it in the Discussion.

13. At the end of the paragraph starting on line 548, the authors say that connections between granular cells and multiple sister cells are not redundant, according to their model. However, the condition on eqn. 14 does not specify this. In fact, all-to-all connectivity would still satisfy eqn. 14, if the weights are adjusted accordingly. Additionally, the prediction that severing these connections would be detrimental to inference is quite generic, and not at all specific to this model.

We thank the reviewer for pointing out the potential for confusion in our use of 'redundant.' By 'non-redundant' we meant that perturbing/removing one of the connections would perturb the computation, not that there be exactly one connection. We agree with the reviewer that our prediction about the effects of severing connections is quite generic, and have removed this from the text.

Minor comments

1. It is confusing that the authors make no distinction between the real causes of an odor and the inferred values of the granule cells. In fact, this distinction is made in Figure 1a, by using x* for the real causes. This should be done throughout the manuscript for clarity.

We thank the reviewer for this suggestion and we have updated the manuscript accordingly, specifically on line 18, Eqn 1 and 3, the paragraph starting at line 52, and line 85. To be consistent with this usage, we now use a subscript of 0 instead of the '*' in Equation 47 to refer to the steady state solution

2. The authors make the point that the average of the sister cells behave exactly like the “original” mitral cell, i.e. that eqn. 15 and eqn. 10a are identical. This is only true if the initial conditions are the same, which needs to be mentioned. In fact, I believe that all simulations start with zero activity, so this condition is trivially met, but the authors should mention it.

The reviewer is correct that for the dynamics to be the same, the initial conditions have to be the same, and we have now updated the text to mention this, on line 163-164. As the reviewer suggests, the Methods now also mention that all simulation start with zero activity (line 788). Note however that the asymptotic solution, which is what we care about, is the same independent of initial conditions.

3. In figure 3d, the last sister cell on the right is missing one of the connections.

We are grateful to the reviewer for this detailed observation. Because the granule cells connect randomly to sister mitral cells, it's generic for the sister cells to have different numbers of connections. We have now updated the caption to avoid confusion.

4. In figure 4, the caption should mention that (I assume) the different shades of green refer to the blocks.

We thank the reviewer for spotting our omission (which we believe refers to Figure 3.) We have now updated the caption to explain the shades of green.

5. In line 197, "The natural coupling is…". This needs to be justified, either by explaining why it is "the natural" one, or by providing adequate references.

The reviewer is correct that "natural" was probably a bad choice for a descriptor. We have now changed that to "reasonable" (line 196).

6. In the simulations, the authors should mention how connectivity was set. Was it randomly selected for each simulation?

We do in fact mention how the connectivity was set in the 'Simulations' section of the Methods: the affinity matrix was set as in Table 1, the sister connectivity was set as in Eqn. 18 and whenever we computed metrics over multiple trials, a new, randomly generated olfactory bulb was used (unless mentioned otherwise). However, the reviewer makes a good point that it should be in the Main Text, and we now do so on line 311.

7. The figures with GC activity would greatly benefit from the inclusion of the real values, i.e. x^*. That way it can be readily evaluated whether the system is performing MAP and whether MAP is accurate at all.

We definitely agree with the reviewer that it is very useful to indicate the MAP solution to allow the reader to readily evaluate whether the system arrives at it. That is why we've used the red triangles on figures showing the time course of responses to show the MAP solution. See for example Figure 5C and its caption. Regarding the comparison to real values x^*: as we believe the reviewer is aware, MAP does not necessarily recover the true odour x^* (see for example, Figure 10A). Since our circuit is designed to perform MAP, we believe (as the reviewer pointed out above) the key comparison is to the MAP solution, rather than the true odour x^*.

8. Figure 10c is referenced in line 292, but does not exist.

Sorry -- we meant 10B. Corrected, thanks!

9. In the sentence starting at the end of line 537: I thought that this only holds with \epsilon = 0. Otherwise, the sister cells have a ratio that can depend on odor. In line 291, the authors mentioned that in their simulations, this dependence on the odor is weak, and point to figure 10c, but there is no figure 10c.

We thank the reviewer for making this point (and apologize for our reference to Figure 10C where we meant 10B). For non-zero epsilon the responses acquire an odour dependence, but as we show in Figure 10B, this dependence is weak. Nevertheless, this odour dependence does complicate our predictions. To address this point, we have now made a new, we hope more robust, prediction: the activity at convergence of any sister cell is a monotonic function of the activity of any of its other sisters. We describe this prediction in the new Section 2.6, and the new Figure 13, where we show numerically that monotonicity as measured by Spearman rank correlation remains very high even at large values of the leak. This new prediction can be tested experimentally. See also our reply to point 3 of Reviewer 2.

10. In line 554, the authors use the word “theory”. Should be replaced by “model” or “hypothesis”.

Thank you, we have replaced it with “model”.

11. In the paragraph starting on line 548, the authors suggest that maximum sparsity would be expected. However, from their results, it seems that there is a trade-off between the number of sister cells and deviations from MAP. In this case, it might be better for the brain to find a good balance between sparsification and accuracy, as opposed to maximally sparsify.

The reviewer is correct that if there is periglomerular leak, more sister cells means slightly higher RMS error, although the effect is small and seems to converge after about 16 sisters (Fig. 10A). However, our statement wasn’t a general one, we said: “if, as we propose, the function of the sister mitral cells is to sparsify the mitral-granule cell connectivity required for inference, then maximally sparse connectivity solutions would be expected,” Because of the “if”, we were explicitly not considering tradeoffs, and the brain may indeed aim to balance sparsification and accuracy, as the reviewer suggests.

Reviewer #2 (Dinu Albeanu)

Tootoonian and colleagues developed a rigorous model for sensory inference aimed at obtaining a maximum a posteriori (MAP) inference solution. Specifically, they are taking advantage of the olfactory circuit architecture to find a solution supported by biologically plausible sparse connectivity. This is in contrast with standard MAP implementations that require dense, biologically unrealistic, all-to-all connectivity. The authors' model assigns different sister mitral cells which receive input from the same common glomerulus the job of extracting different latent variables and posits that each sister cell connects reciprocally and symmetrically to different sets of inhibitory granule cells interneurons. This strategy enables each mitral cell to interact with a smaller number of granule cells (by a factor S, ~25 given the number of sister cells per glomerulus). They further achieve coordination among sister mitral cells, necessary for their model implementation to arrive to MAP solution, by engaging back propagating action potentials from the sister mitral cells to drive the firing of periglomerular cells which, in turn, inhibit the sister mitral cells and drive them to converge at equilibrium to the same activity patterns, or at the same ratio in an odor independent fashion (a strong prediction of the model). In the current implementation, the readout units are the granule cells, which nevertheless do not relay information directly to the rest of the brain. The authors argue that a similar architecture with matched weights can be implemented at the level of pyramidal cells in the cortex that can also extract the same kind of information w/o having to relay it back to the mitral cells. The model solves the inference problem successfully for a small number of odors in the sensory scene, but its performance becomes poorer and poorer with increasing number of odors within the range sampled (up to 10). In my opinion, model is elegant and clearly spelled out, the manuscript is well-written, with numerous explanations of the assumptions and possible caveats and solution, and, thus, a pleasure to read. I also found the idea of using the sister cells as means to implement the necessary sparse connectivity very clever. However, my enthusiasm was reduced by the poor performance of the model in conditions where the number of odorants to represent is relatively small (>3-4), as well as by several implementation choices and its predictions that, in my opinion, depart from known biological evidence. I list below my main concerns:

1. No detail/reasoning is provided with respect with how the sparse prior is stored in the brain/model architecture. How is the prior information represented, stored and how is it acquired?

We thank the reviewer for pointing out this oversight in our explanation. In our model the prior information is represented in the threshold and gain of the GC firing rate function (compare the prior parameters beta and gamma in equation 6 with the granule cell firing rate function in Eqn 33d). These can be set on evolutionary timescales as the animal adapts to its environment. Cortical feedback can also adjust these by, e.g., providing input current that effectively modifies the threshold parameter beta. We have now updated the text after Equation 33 to mention these points.

2. The model does not seem to work efficiently for simple sensory scenes where multiple odorants are present. It appears that starting from n=3 odors, the model starts to recover many more odors than are actually present.

We apologize for the confusion about this point. The apparent poor performance of the model is simply due to the fact that we simulated systems with a small number of glomeruli (small M), to save time. Increasing M increases the maximum value of n that MAP can correctly infer, as we demonstrate in the updated Figure 11 (see panel B). We also now mention this in the paragraph 'Number of odour components' in the Main Text, lines 409-410.

3. The hard prediction of the model that all sister cells converge to the same activity is **not** necessarily supported by previous experimental work in anaesthetized and awake mice (Dhawale et al., 2010, Arneodo et al., 2017) that identified sister mitral cells in vivo and showed that their activity patterns are non-redundant and, if anything, diverge in time. Average firing rate changes of sister cells tend to be indeed somewhat similar to each other (more so than pairs of non-sister cells), but they span a wide range. Furthermore, the ratio of their responses appears to not odor-independent, but to vary as function of the odor presented. Their average odor responses at steady state are not simply scaled versions of each other, displaying instead a wide distribution of response correlations. Throughout the manuscript, the predictions of the model with respect to sister mitral cells converging to same activity patterns at equilibrium and whether they have or not the same ratio of response to different odors vary across different sections. With respect to their spike-timing, experimental work found that sister cells become decorrelated in an odor specific manner, both with respect to each other, as well as with the respiration clock. The model does not address these biological results (in the current implementation, it does not discuss the transmission of information with respect to different phases of the respiration cycle).

We apologize for the lack of clarity in our writing. We did indeed make multiple predictions. It's true that each one was qualified, but we did not emphasize those qualifiers nearly enough. We now make only one prediction about the activity of sister cells at convergence, in the new Section 2.6, "Relative sister cell activity at convergence" and associated Figure 13: if we show multiple odors, plot the responses of any two sister mitral cells against each other in response to those odors, those points should be monotonic (see, for example, Fig. 13B,C) -- something that could be tested experimentally.

Is this prediction inconsistent with current data? The results in (Arneodo et al. 2017) cannot be directly compared to our predictions because they do not report results from two sisters in the same animal. We looked at Dhawale et. al 2010 (which inspired this work!), and Fig. 5C shows the responses of sister mitral cells to three odors. We estimated the responses of the sister mitral cells to odours by counting spikes in the odour window, and assuming the response of unit 1 to 2-heptanone is larger than the response to heptanol, then the sister cells do have a monotonic relationship, as predicted (see the table below; note also that the effect is stronger if we subtract the baseline). This is not strong evidence for our model, but it is not evidence against it.

**Odour**	**Unit 1 response**	**Unit 2 response**
heptanal	4	0
2-heptanone	5	2
p-anis aldehyde	20	12

The reviewer also mentioned that our predictions varied throughout the original manuscript, so we hope that by focusing our Discussion of predictions to a clearly marked section of the text and to a dedicated figure, it is now clear exactly what our predictions are and how they may be experimentally tested.

4. The authors choose to not implement cortical feedback in this version of their model (though in previous work they have, Grabska-Barwinska et al., 2017), which constraints its relation to the biology, since a cortical feedback is a key feature of the vertebrate olfactory circuit. For example, firing of the granule cells is heavily dependent of cortical input and controls the timing and gain of mitral cell firing. Also, cortical feedback may be important in relaying the required sparse prior.

The reviewer is correct that cortical feedback is a key feature of the vertebrate olfactory system. Our model is in a certain sense agnostic to how the granule cell activations are implemented, whether directly through the mitral cells as we have now, or via a disynaptic pathway in which mitral cells excite cortical neurons which then excite the granule cells. Because the effect of cortical feedback was outside of the scope of this work and is not required to relay the sparse prior (see reply to point 1), we chose the first implementation for simplicity.

5. As the authors note, the outputs of individual glomeruli consist of both mitral and tufted and sister cells with very different intrinsic properties (including substantially different firing properties), local wiring and projection patterns. However, in the current implementation both populations are bundled together. This is to some degree a technical detail, but it does have important consequences on key requirements/benefits of the model: whether it is realistic to consider convergence to similar firing patterns in time (this appears different for tufted and mitral cells) and the size of S (Schwarz et al., 2018).

The reviewer is of course correct that MCs and TCs differ anatomically, biophysically and functionally, and are likely to play different roles in olfactory bulb computations. Because our model describes how a *given* computation can be spread among sisters to reduce connectivity requirements, and MCs and TCs perform different computations, then our predictions would only apply to sister MCs, and separately to sister TCs. We now mention this important point at the end of the 'Predictions' section of the Discussion. See lines 528-536.

6. The choice of periglomerular cells activated by back-propagating action potentials of mitral cells as a source to regulate the similarity of sister cells is interesting, but further discussion would be needed in terms of the assumptions made with respect to connectivity within the glomerulus of these cells and their timing of spiking with respect to the firing of mitral cells. The activity profiles of mitral, periglomerular and granule cells shown in Figs. 9 & 12 appear quite different from documented activity patterns of such cells in the brain and converge to a stable steady state within 100-200 ms from stimulus onset.

Thank you for making this important point. Generally, we agree that the convergence of activity to the stable states we show in e.g. Figs 5,6 and 9 is not what is typically observed in experimental settings. However, we have not implemented realistic features like rhythmic sampling (respiration), or included additional cell types (such as slow Ca2+ spike generating JGCs or intrinsically rhythmic ET cells etc) that would add to the complexity of longer-term temporal dynamics. We believe that adding these further biological details - while possibly useful in the future - would significantly distract from the core result of our paper, namely that sister cells are a plausible implementation of sparse MAP inference with PGCs providing a measure of the average activity per glomerulus. We have added lines 216-220 to highlight the existing literature on PGCs in relation to the requirements of our model.
