# Supplementary material for: Sparse connectivity for MAP inference in linear models using sister mitral cells
Source: PLoS Comput Biol. 2022 Jan 31;18(1):e1009808. doi: 10.1371/journal.pcbi.1009808 (PMC8830798; doi:10.1371/journal.pcbi.1009808)
Supplement: Attachment — Submitted filename: point-by-point-r2.pdf [file pcbi.1009808.s002.pdf]

Point-by-point reviewer comments and responses

Reviewer #1

The authors have addressed most of the major and minor points from the previous round. However, one important point remains unaddressed...perhaps the authors meant to introduce an arbitrary model (eqn 10) and then show that it performs MAP, just as the system in eqn 9 does. While it might sound like a distinction without a difference, it is important to note that equations 9 and 10 do not describe the same system at all, but do converge to the same equilibrium point. If this was the authors' intention, please indicate so in the manuscript, especially in the transition from equation 9 to 10.

We thank the reviewer for their rereading of our manuscript, and apologise for the confusion caused. The reviewer's guess is correct: we did not mean that the transition from Eqn 9 to 10 is a change of variables, rather we wanted to motivate the new system in Eqn 10, which as the reviewer points out is different to that in Eqn 9 but has the same fixed point. We have now explicitly indicated that the two systems are different but have the same fixed point (line 124).

For completeness, a condition of continuity could be added to the transformations f, such that invertible implies monotonic (as said in line 435).

We in fact require the transformations to be not just continuous, but differentiable, as we take partial derivatives in Eqn 37. We have now explicitly added this differentiability condition to the transformations f (lines 434 and 437).

Reviewer #2

The authors have addressed several of my concerns and substantially improved the manuscript which, I think, will have an important impact in the field. I could still quibble with the exact implementation schemes (no cortical feedback) and the convergence of sister mitral cells (happy to share the data on their odor response spectra), but, in my opinion, these points could be addressed in further manuscripts, and this one is now ready for publication.

We thank the reviewer for reading our manuscript again, and certainly concur that the role of cortical feedback and the convergence of sister cells are very important points. Given the size of the current manuscript, we agree with the reviewer that these points can be addressed in future work.
